# The Performance Implication of Corporate Social Responsibility: The Moderating Role of Employee’s Prosocial Motivation

**DOI:** 10.3390/ijerph18063128

**Published:** 2021-03-18

**Authors:** Min-Jik Kim, Byung-Jik Kim

**Affiliations:** 1 School of Industrial Management, Korea University of Technology and Education, 1600, Chungjeol-ro, Byeongcheon-myeon, Dongnam-gu, Cheonan-si 31253, Korea; mkim@koreatech.ac.kr; 2 College of Business Administration, University of Ulsan, Ulsan 44610, Korea

**Keywords:** corporate social responsibility, organizational performance, organizational commitment, prosocial motivation, moderated mediation model

## Abstract

Although there has been extensive research on the corporate social responsibility (CSR)–performance link, full understanding is still elusive. A possible reason for this is the limited understanding of the underlying processes that affect the relationship. Grounded in institutional theory, which emphasizes the importance of micro-level intermediating processes (e.g., employees’ perceptions and attitudes) to explain a macro-level association (i.e., CSR to organizational performance), we built a moderated mediation model where: (i) organization commitment mediated the influence of CSR on organizational performance, and (ii) an employee’s prosocial motivation moderated the relationship between CSR and organizational commitment. Using three-wave time-lagged survey data obtained from 302 Korean workers, we found that organizational commitment is an important micro-level process in the CSR–performance link, and that the level of an employee’s prosocial motivation can positively moderate that link. We discuss theoretical and practical implications, along with limitations and future research directions.

## 1. Introduction

In recent decades, corporate social responsibility (CSR) has become an increasingly important issue in the management literature and business fields. As firms allocate more resources for CSR, researchers and practitioners have paid greater attention to the effect of CSR on organizational performance (OP). For example, some scholars have reported that CSR plays a strategic role to facilitate the competitive advantage of companies [1,2,3,4]. However, others have found that the relationship is not significant and may even be negative [5,6,7]. These inconclusive results call for more work on the CSR–performance link. Against this backdrop, some previous studies [8,9,10,11,12,13,14,15] have suggested that future work on CSR needs to elaborate on the underlying processes (e.g., mediators and moderators) of the relationship to resolve the inconclusiveness.

Thus, we consider some research gaps in previous studies on the CSR–performance link. First, there is still a need to explore the influence of CSR on internal stakeholders (i.e., employees) because this is relatively less explored. Although the employee is the main agent who actually implements CSR practices [13,15], previous studies dealing with the effect of CSR on performance have mostly concentrated on the relationship between CSR and external stakeholders such as shareholders, customers, suppliers, and governments [16,17]. However, the perceptions, attitudes, and behaviors of employees are important in explaining or predicting the impact of CSR activities on performance [18,19]. An internally focused approach may help to extend our understanding of the CSR–OP link by complementing the previous externally focused perspective.

Second, previous works have relatively ignored the importance of the employee sense-making process to react to CSR practices [13,15]. Considering that not only may all employees in an organization not perceive and respond to the organization’s CSR practices in the same way, but also the sense-making process or interpretation of each employee is likely to determine his or her perceptions and reactions toward moral practices (i.e., CSR activities), we suggest that the employee’s sense-making procedure or interpretation may function as a critical contextual factor (i.e., moderator) by substantially influencing his or her perceptions and attitudes towards CSR activities.

To fill the gap in the literature, we adopt an approach that incorporates internally and externally focused perspectives. Grounded in institutional theory, which emphasizes the critical role of micro-level mechanisms (e.g., employee’s perceptions, attitudes, and behaviors) in the association between macro-level variables (e.g., an organization’s structures, systems, outcomes, and practices, such as CSR activities and organizational performance) [20], we investigate an underlying intermediate mechanism that translates CSR into OP. According to the theory, the micro-level processes may mediate the association between the macro-level variables [20]. Specifically, this paper focuses on organizational commitment (OC) as a mediator between CSR and OP. Organizational commitment can be defined as the level of an employee’s psychological attachment to, as well as their will to, endeavor to achieve his or her organization’s goals [21,22]. Because employees are likely to not only highly value, but also be proud of their firm’s CSR activities, the level of an employee’s commitment to their organization would be increased [23,24,25,26,27,28,29]. Further, OC has been known to function as a fundamental attitude toward an organization because it is closely associated with a variety of organizational outcomes, such as job satisfaction, turnover, in-role/extra-role performance, and OP [30,31,32,33,34]. Thus, the elevated level of OC promoted by CSR may improve OP, and we argue that OC is a critical mediator in the association between CSR and OP. By demonstrating the mediating role of an employee’s OC, this paper may reveal the important function of a micro-level intermediating mechanism to explain the macro-level association.

More importantly, we expect that an employee’s prosocial motivation may play a role as a critical moderator in the relationship between CSR and OC. Prosocial motivation can be defined as the desire to expend effort to benefit other people [35,36]. This concept includes someone’s desire to protect and promote the welfare of other people and being facilitated by contact with other people in need of help [35,36]. When an employee has a high level of prosocial motivation, they may positively and favorably respond to CSR activities, as the moral practices of the organization closely correspond with the values they pursue [37,38,39]. Then, the positive impacts of CSR activities on his or her perceptions and attitudes towards the organization (i.e., OC) would be enhanced. However, when the employee does not have a high enough level of prosocial motivation, they may devalue the moral attempts of the organization (i.e., CSR activities) by not actively cooperating with the practices or even disregarding them because they do not care about achieving moral values. Then, the positive effect of CSR on OC would be weakened. Thus, we argue that the degree of an employee’s prosocial motivation would moderate the CSR–OC link. This attempt may contribute to CSR literature by revealing the elaborate contextual or contingent factor in explaining the CSR–OP link.

Taken together, we believe that this paper may contribute to CSR literature in several ways as follows. First, we emphasize the important role of micro-level mechanisms (i.e., OC and prosocial motivation) in the association between macro-level variables (i.e., CSR and OP). Second, by empirically demonstrating that an employee’s prosocial motivation functions as a critical contextual factor to explain the CSR–OP link, this paper reveals that the sense-making process of employees (i.e., prosocial motivation) may determine their reactions toward CSR activities. Lastly, to empirically test our hypotheses, we examine the underlying mechanism with a moderated mediation model by using three-wave time-lagged data.

## 2. Theory and Hypotheses

### 2.1. CSR and Organizational Commitment

Previous works have reported that CSR activities may facilitate the degree of employees’ OC [23,24,25,26,27]. Because CSR practices may provide valuable programs that employees appreciate, it is likely to affect employees’ positive attitudes, such as their commitment to the organization. Thus, the literature on the CSR–OC relationship has primarily relied on social exchange theory, which explains a mechanism of continuous exchanges between different parties [28,29].

The social exchange theory has been known to be grounded on a reciprocity principle. If someone provides something precious for the beneficiary, he or she may think that he or she has a kind of duty to repay it in a similar way or at a similar level [28,29]. For instance, if a firm provides a variety of benefits for its stakeholders, such as employees, customers, their community, and the environment, the stakeholders are likely to perceive a sense of obligation to repay it. From the perspective of a firm, its members function as employees, consumers, and members of the community, and so they may benefit directly and indirectly from CSR activities [28,29]. Therefore, the employee is likely to perceive that he or she must do it for the firm. One way that employees repay is that they show positive attitudes and behaviors such as commitment to the organization.

**Hypothesis** **1.** *CSR is positively related to OC.*

### 2.2. OC and OP

We also argue that a higher level of OC ultimately contributes to OP. OC is a critical concept in an organization because of its close relationship with important organizational outcomes such as job satisfaction, turnover, in-role/extra-role performance, and OP [30,31,32,33,34]. Specifically, existing meta-analyses have reported that OC is associated with OP, which is one of the most critical goals in an organization [32,33,34].

Employees who have a high degree of OC may perceive and consider the aims, values, and interests of their organization as their own. Therefore, they are likely to make sincere efforts to fulfill the expectations of the organization by working harder. Then, those efforts are transformed into the form of enhanced in-role/extra-role performance by increasing the quality of employees’ attitudes and behaviors [32,33,34]. This enhanced OC is likely to be shared among employees in the organization through a social contagion process [40]. In turn, the shared OC may be summarized at the collective level as forming a “common attitudinal ground” [41].

If collective OC is created via social interactions in an organization, members may feel that they not only want to be loyal to the organization, but also to invest their cognitive, emotional, and physical resources to realize the organization’s goals [40,41,42]. Then, employees tend to help their colleagues achieve these aims by providing an increased level of cooperating behaviors. These behaviors then boost the quality of interactions in the process of conducting various work-related tasks, facilitating morale and cohesiveness in an organization. Eventually, the enhanced attitudes and behaviors would directly contribute to increasing organizational effectiveness and performance [40,41,42].

**Hypothesis** **2.** *OC is positively related to OP.*

### 2.3. The Mediating Effect of OC in an Association between CSR and OP

As mentioned, grounded in institutional theory (which is our overarching framework), we argue that the impact of CSR on OP would be mediated by employees’ OC. Institutional perspective [20] suggested that various organizational practices, structures, and systems (e.g., CSR activities) as institutional enablers tend to affect employee’s perceptions, attitudes, and behaviors, which are micro-level underlying mechanisms. In turn, the employees’ reactions in the form of perceptions, attitudes, and behaviors significantly influence various institutional outcomes, such as organizational performance. Thus, we expect that the effect of CSR on OP can be mediated by the micro-level variables of employees’ OC. In other words, OC may mediate the CSR–OP link.

**Hypothesis** **3.** *CSR influences OP through a mediation of OC.*

### 2.4. The Moderating Effect of Prosocial Motivation in the CSR–OC Link

As mentioned above, the relationship between CSR and OC has been theoretically and empirically validated in previous works [23,24,25,26,27]. However, it seems too simple and naive to believe that CSR will always increase OC in all situations and conditions, as members tend to react differently to CSR practices.

Choosing from several factors that influence members’ responses to CSR, this paper focuses on the individual characteristics of the member. Given that a member in an organization is likely to not only try to make sense of their work experiences, but also actively interact with their organization [43,44], we can reasonably expect that employees may not passively accept and follow the CSR activities of the firm. Being grounded in their interpretation from the sense-making procedure, they would determine how to react and cooperate with the moral practices of the firm (i.e., CSR). To be specific, the interpretation of an employee would significantly moderate the influence of CSR on OC.

Among a variety of potential variables that affect sense-making and interpretation procedures, we concentrate on the motivational characteristics of each member, because the motivational features are closely associated with the sense-making process by directly regulating his or her perceptions, attitudes, and behaviors [36,45,46]. Additionally, to maximize the conceptual compatibility with CSR practices that pursue moral values, we investigate the interactive effect of the prosocial motivation of an employee with CSR activities. Prosocial motivation indicates the desire to expend effort to benefit other people [35,36]. From the conceptual perspective, CSR activities and prosocial motivation have moral features in common by pursuing ethical values. Thus, we expect that prosocial motivation may substantially influence the employees’ sense-making processes and interpretation of the firm’s CSR activities, eventually determining their reactions (e.g., perceptions, attitudes, behaviors) toward moral practices (i.e., CSR activities).

Specifically, an employee with a high level of prosocial motivation is likely to want to contribute to society, so they may perceive that the moral practices of the organization closely correspond with the values they pursue. Then, they would favorably respond to the CSR activities. According to person–organization fit theory [37,38,39], synergy effects tend to occur when an individual employee and the organization have the same or similar value systems. In an organization that conducts moral activities such as CSR, if its members have a high level of prosocial motivation, the suitability of the organization and the individual is very high. In this situation, the employee would recognize that the CSR practices satisfy their moral needs to contribute to society [38,39]. Then, the employee is likely to positively respond to the CSR by boosting their commitment to the organization.

However, an employee with a low degree of prosocial motivation may not empathize with the moral values that the organization pursues. Because the employee does not want to achieve the moral values, they are likely to devalue the moral attempts of the firm by not actively cooperating with the practices, or even disregarding them. No matter how enthusiastically the organization implements CSR activities, if the employee has a low level of prosocial motivation, they are likely to be indifferent or even respond negatively to the CSR because their suitability to the organization is low. If so, the positive impacts of the moral practices would be diminished, reducing the positive impact of CSR on OC.

**Hypothesis** **4.** *Employee’s prosocial motivation would positively moderate the CSR–OC link.*

**Figure 1:**
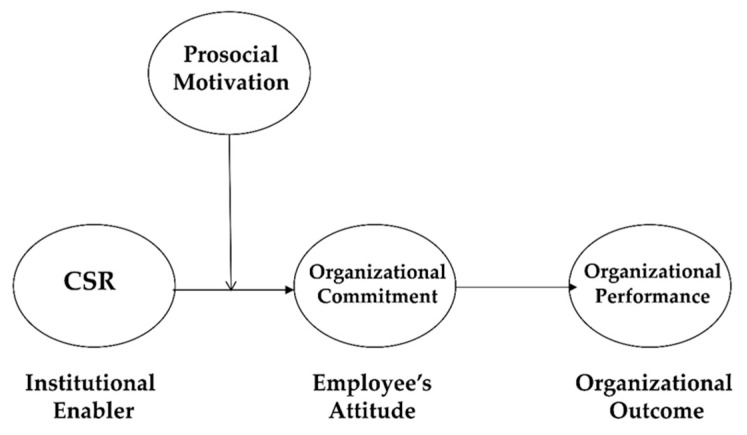
Theoretical model. CSR: corporate social responsibility.

Taken together, we propose that CSR enhances OP through a mediating effect on OC. Moreover, prosocial motivation positively moderates the CSR–OC link (Please See Figure 1).

## 3. Method

### 3.1. Data Gathering

By using an online survey system, we gathered data from workers who were working in South Korean firms during three distinct time points. To decrease the harmful effects of sampling bias, we used a random sampling method. Through gathering data at 3 distinct time points and from distinct sources (e.g., employees and the director of the human resource management (HRM) department in each company), we tried to resolve the limitations embedded in cross-sectional data.

At Time Point 1, 512 workers participated in the survey. At Time Point 2, 378 workers participated in our second survey, and at Time Point 3, 335 workers participated in the last survey. The interval between each time point was about 4 or 5 weeks. Our survey system was opened for about 2 or 3 days to provide enough time for the respondents to complete the survey. During these times, they could freely access the system. After that, we eliminated incomplete and missing data from the raw dataset. Finally, this research used data from 302 employees for the final analysis (response rate: 58.98%). The features of the respondents are described in Table 1.

### 3.2. Measures

We measured our variables with a 5-point Likert scale that ranged from one to five (one is strongly disagree, five is strongly agree).

#### 3.2.1. Corporate Social Responsibility (Time Point 1, Gathered from Employees)

CSR practices were evaluated by employees using the 12-item Turker’s CSR scale [47]. The scale tends to take the stakeholders’ perspectives into account, consisting of items about various CSR activities for a variety of stakeholders. The scale has four dimensions: (a) CSR for the environment, (b) community, (c) employee, and (d) customer. To evaluate the degree of CSR practices for the environment, we used three items, such as “our firm participates in activities which aim to protect and improve the quality of the natural environment.” We evaluated CSR for the community with three items, including “our firm contributes to campaigns and projects that promote the well-being of society.” Third, we evaluated CSR for the employee with three items, including “the management of our company is primarily concerned with employees’ needs and wants.” Finally, we used three items to evaluate the degree of CSR for the customer, such as “our firm respects consumer rights beyond the legal requirements.” The Cronbach’s alpha was 0.90.

To evaluate whether the CSR measures included the four-dimension structure, we performed a confirmatory factor analysis (CFA). Then, we performed chi-square difference tests in a sequential manner to compare the fit indices of our four-factor model to other alternative models (i.e., three-factor, two-factor, and one-factor models, respectively). This paper utilized the comparative fit index (CFI), the Tucker–Lewis index (TLI), and the root mean square error of approximation (RMSEA) as criteria for evaluating the fit indices. The results of the difference tests showed that our four-factor model (χ^2^ (df = 45) = 86.978; CFI = 0.977; TLI = 0.966; RMSEA = 0.056) was better than the other models. 

#### 3.2.2. Organizational Commitment (Time Point 2, Collected from Employees)

At Time Point 2, to measure OC, we used four items of the organizational commitment scale of Meyer and Allen [22]. Sample items are: (a) “I really feel as if my organization’s problems are my own,” (b) “I feel a strong sense of belonging to my organization,” and, (c) “I feel emotionally attached to my organization” (Cronbach’s alpha = 0.87).

#### 3.2.3. Organizational Performance (Time Point 3, Gathered from the Human Resource Management Director of Each Company)

The director of the HRM department in each firm was evaluated regarding the degree of OP through four items (Cronbach’s alpha = 0.92). Sample items are from previous research [48] and consisted of: “Our firm is more efficient and productive than our competitors,” “Our management performance is superior to our competitors,” and “Our financial performance is excellent in comparison to our competitors.” By gathering data from different sources, we tried to diminish the potential harms of common method bias.

#### 3.2.4. Prosocial Motivation (Time Point 1, Collected from Employees)

To measure employee’s prosocial motivation, we used five items from Grant and Sumanth [49]. Sample items are: “I get energized by working on tasks that have the potential to benefit others,” “It is important to me to have the opportunity to use my abilities to benefit others,” and “I prefer to work on tasks that allow me to have a positive impact on others.” The value of the Cronbach’s alpha test in this study was 0.85.

#### 3.2.5. Control Variables

OP was controlled by company size and the industry type of each firm, and OC was controlled by employee’s tenure (in months), gender, position, and educational level [50,51]. To maintain the consistency of this study, we collected the control variables at Time Point 2.

### 3.3. Analytical Strategy

The associations among the variables were evaluated by a Pearson correlation analysis. As suggested by Anderson and Gerbing [52], a two-step approach that consists of the measurement and the structural model was applied. To check the validity of the measurement model, we performed a CFA for our research variables. Then, we performed structural equation modeling (SEM) analysis by building a moderated mediation model to test our structural model. We used the maximum likelihood (ML) estimator to perform the SEM. Moreover, to evaluate our mediation hypothesis, we performed a bootstrapping procedure by using the 95% bias-corrected confidence interval (CI) to evaluate the mean indirect mediation. If the CI excludes 0, it is interpreted that the indirect effect is statistically significant at a level of 0.05.

To test if the model fits were adequate, we used a variety of goodness-of-fit indices, including the comparative fit index (CFI), the Tucker–Lewis index (TLI), and the root mean square error of approximation (RMSEA). Extant studies reported that an adequate fit is indicated by CFI and TLI values over 0.90 and an RMSEA value less than or equal to 0.06 [53]. Lastly, a bootstrapping analysis was performed to check if the indirect effects were significant [54].

## 4. Results

### 4.1. Descriptive Statistics

The result of the correlation analysis is described in Table 2. The study variables, including CSR, ethical leadership, OC, and OP, were significantly associated.

### 4.2. Measurement Model

We conducted a CFA for all 21 items to check the goodness-of-fit of the measurement model. Because our three psychometric constructs from the same employee (i.e., CSR, OC, and prosocial motivation) were included in the model, the discriminate validity of the three variables was checked. The three-factor model showed a good fit (χ^2^ (df = 59) = 93.835; CFI = 0.982; TLI = 0.976; RMSEA = 0.044). Additionally, we performed a series of chi-square difference tests by consequently comparing the three-factor model to two-factor (χ^2^ (df = 61) = 602.927; CFI = 0.715; TLI = 0.635; RMSEA = 0.172) and one-factor (χ^2^ (df = 62) = 733.646; CFI = 0.646; TLI = 0.555; RMSEA = 0.190) models. The results of the chi-square difference tests indicated that the three-factor one was best. Therefore, we suggest that the three variables have an adequate level of discriminant validity.

### 4.3. Structural Model

In this paper, we built a moderated mediation model that contains both mediating and moderating structures between CSR and performance. In the mediating structure, the CSR–performance link was mediated by OC. Moreover, in the moderating structure, prosocial motivation plays a moderating role in the CSR–OC link.

To identify if there is a multi-collinearity bias between our independent variables (i.e., CSR and prosocial motivation), we checked the variance inflation factors (VIF) and tolerances [55]. The VIF value for both CSR and prosocial motivation are 1.09 and 1.09. In addition, the tolerance value for both variables are 0.92 and 0.92. Because the VIF scores are smaller than 10, and the tolerance scores are above 0.2, we suggest that CSR and prosocial motivation are relatively free from the problem of multi-collinearity.

#### 4.3.1. Results of Mediation Analysis

We performed SEM analyses and a chi-square difference test between the full mediation model and the partial mediation model. The model fit of our full mediation model was good (χ^2^ = 248.013 (df = 137); CFI = 0.957; TLI = 0.941; RMSEA = 0.052). A partial mediation model includes a direct path from CSR to performance. The model fit of our partial mediation model was adequate (χ^2^ = 245.534 (df = 136); CFI = 0.958; TLI = 0.941; RMSEA = 0.052). The result of our chi-square difference test between the models demonstrated that the full mediation model was better (Δχ^2^ [1] = 2.479; *p* > 0.05 (non-significant)).

**Figure 2:**
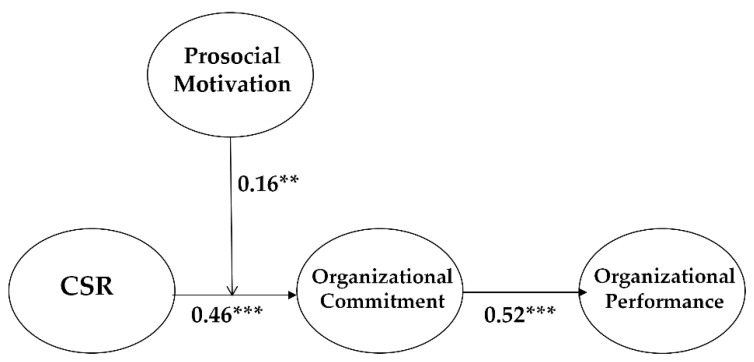
Coefficient values of our research model. ** *p* < 0.01, *** *p* < 0.001

Figure 2 shows the final model. The control variables, including gender, position, and firm size, were statistically non-significant. Including the control variables, the final model demonstrated that CSR is significantly related to OC (β = 0.46, *p* < 0.001), thus supporting Hypothesis 1, and that OC is significantly associated with OP (β = 0.52, *p* < 0.001), thus supporting Hypothesis 2.

#### 4.3.2. Results of Moderation Analysis

The moderation influence of prosocial motivation on the relationship between CSR and OC was evaluated by the moderated mediation model. To make an interaction term, we conducted a mean-centering procedure. Centered variables have been known to not only estimate the interaction terms in an efficient way, but also diminish multi-collinearity among the variables [55].

**Figure 3:**
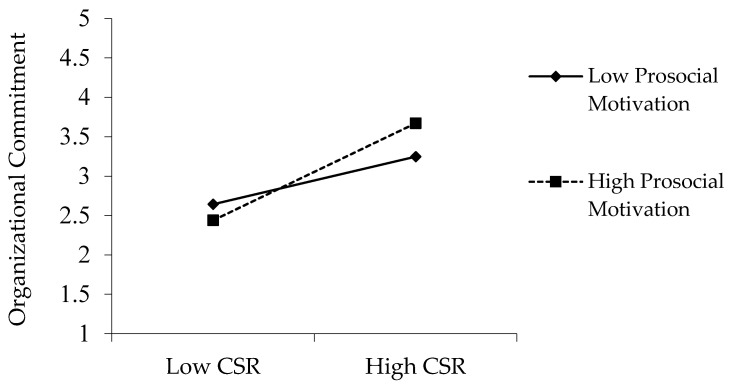
Moderating effect of prosocial motivation in the CSR–OC link.

The coefficient value of the interaction term (β = 0.16, *p* < 0.01) was statistically significant, indicating that prosocial motivation positively moderates the CSR–OC link. This means that when the degree of prosocial motivation is high, the positive influence of CSR on OC is increased, supporting Hypothesis 4 (Please See Figure 3).

### 4.4. Bootstrapping

The bootstrapping technique was used with a sample of 10,000 [54] to evaluate Hypothesis 3, which suggests the mediation effect of OC in the CSR–performance link. The effect of indirect mediation was significant at a 5% when the 95% bias-corrected confidence interval (CI) for the mean indirect mediation excluded zero [54]. The bias-corrected CI for the effect excluded zero (95% CI = [0.15, 0.52]). Therefore, this result indicates that the indirect mediation effect of OC on the CSR–performance link is statistically significant at 5%. This result supports Hypothesis 3.

## 5. Discussion

Using three-wave time-lagged data, we revealed that employees’ OC functions as an intermediating factor in the association between CSR and OP. We also found that employees’ prosocial motivation positively moderates the CSR–OC link. Our research can be differentiated from existing studies because we concentrated on the internal mechanisms of CSR by investigating mediating and moderating factors that explain why and when CSR may enhance OP. In the following section, we discuss the theoretical and practical implications of our work. We also point out the limitations and suggest some possible future directions.

### 5.1. Theoretical Implications

We believe that this paper may positively contribute to the CSR literature for two reasons. First, this research attempted to integrate the macro- and micro-approach in explaining how CSR influences OP. By demonstrating that micro-level mechanisms (e.g., employees’ attitudes, such as OC) can function as an intermediating factor in the macro-level association (e.g., CSR and OP) [56], the results of our research extend the CSR literature by applying the integrating perspective. While previous works have mainly focused on the relationship between CSR and external stakeholders, we provided a micro-level underlying mechanism that originated in the internal stakeholders to elaborately explain the association. This approach would complement the previous externally focused approach.

To be specific, based on the institutional theory, this paper suggested and demonstrated that CSR activities, as a critical institutional enabler, build employee’s positive reactions toward his or her organization in the form of increased OC, eventually boosting the level of organizational outcomes (i.e., organizational performance). This indicates that CSR activities can be the active ‘investment’ from the perspective of the firm beyond just a kind of ‘cost’.

Second, this paper showed that an employee’s prosocial motivation functions as a critical contextual factor to explain the CSR–OP link. Considering that the sense-making process or interpretation by the employee is likely to determine their reactions toward moral practices (i.e., CSR) [43,44], we expect that the employee’s motivational characteristics, such as prosocial motivation, directly influence the implementation of various practices, such as CSR, on their perceptions, attitudes, and behaviors. This means that the degree of an employee’s prosocial motivation would moderate the impact of CSR on OC. We demonstrated that the positive influence of CSR on OC is strengthened by a high level prosocial motivation of an employee. The results indicate that the level of an employee’s prosocial motivation should be regarded as an important contingent factor in translating moral endeavors into enhanced employee attitudes toward their organization.

### 5.2. Practical Implications

The current work supplies practical, meaningful implications for leaders in an organization. First, the leaders and top management teams whose organizations are planning to implement CSR activities need to consider their employees as important agents who can improve OP by substantially planning, initiating, and implementing CSR practices. The top management team especially should acknowledge that the reactions of employees in the form of various perceptions, attitudes toward their organization, and behaviors play a critical role in translating moral efforts (i.e., CSR activities) into organizational outcomes (i.e., performance). Therefore, leaders should try to communicate and interact with their employees about CSR practices. In addition, they also have to supply adequate rewards for the employees to maximize active participation through them. By regularly communicating about the organizations’ CSR activities, the top management team can build a CSR-oriented culture within the organization [57].

Second, we suggest that top management teams can better understand the critical role of an employee’s motivational characteristics (i.e., prosocial motivation) by implementing CSR practices. Considering that employees’ sense-making processes or interpretations of the organization’s CSR activities functions as a critical contextual factor by directly influencing the effects of CSR on his or her perceptions and attitudes, the level of prosocial motivation of an employee would critically influence the effects of CSR on OC. If the level of his or her prosocial motivation is low, the positive influence of the moral practices on employees’ attitudes toward their organization would be eroded. In other words, top management teams should understand and acknowledge that the level of an employee’s prosocial motivation functions as a practical gauge to predict whether CSR practices may positively influence their OC or not. Based on our results, we propose that leaders not only have to cultivate their prosocial motivation, but also adopt employees who have a high level of prosocial motivation.

### 5.3. Limitations and Suggestions for Future Studies

The following limitations of our research should be addressed to positively contribute to future works. First, cultural differences between Eastern and Western societies may affect how employees perceive the CSR activities of organizations. Western cultures are more likely to emphasize the importance of socially prescribed obligations such as CSR; thus, the employees may be more sensitive to those social duties [58,59]. Because we collected data from employees of South Korean firms, cautious interpretation of the results is required when the findings are applied in the context of different cultural backgrounds [58,60]. Despite the universality of the spirit of CSR [59,61], Korean employees may have responded differently to the call for the social responsibilities than employees in Western cultures. Thus, further studies on the CSR–OP link should consider cultural differences.

Second, we could not use full items for measuring OC due to some practical constraints in the data collection process. Although we selected the core items of each scale according to the suggestions from previous literature, this may have caused a decrease in construct validity. Nevertheless, both our reliability test and sequential chi-square comparison tests in our research showed sufficient discriminant validity of the constructs. We suggest that future studies should use full items for the OC scale.

Third, we could not include objective indices for measuring CSR. Although previous studies on CSR have suggested that subjective measures, such as employees’ perceptions about CSR, can adequately reflect the real phenomena about CSR in a more accurate way than objective CSR measures [62], we recommend future works to include both subjective and objective CSR measures in its research model.

Fourth, this paper could not sufficiently deal with the potential problem of endogeneity and its remedies. The endogeneity issue tends to originate from various unobservable factors, such as company, CEO, and employee characteristics [63]. For example, CSR, employee’s CSR orientation, and performance are likely to be significantly influenced by various unobservable factors, including firm ownership, culture, reputation, and visibility [64]. Future studies should consider and adequately deal with this issue.

Fifth, this paper only investigated the mediating role of OC in explaining the CSR–OP link. However, it is important to consider that various important micro-level variables (e.g., meaningfulness of work, work engagement, motivation, thriving, vigor, and psychological well-being) are likely to function as a critical intermediating channel to translate the positive influence of CSR activities into organizational performance [8,9,10,11,12,13]. In future studies, this issue should be fully dealt with.

Sixth, we could not fully explore the significant influence of organizational cultures on the perceptions of employees on CSR activities [65,66]. Considering that various organizational cultures, such as ethical culture, caring culture, and supportive culture, may critically shape the level of both CSR activities and employee’s perception on CSR [65,66], this paper may have a critical limitation. Thus, future research should investigate the roles of organizational cultures in explaining CSR practices.

Lastly, prosocial motivation and CSR were collected from the same employees at the same time. Thus, we could not exclude the possibility of bias in the conceptual and empirical overlap between the two variables. An employee with a high level of prosocial motivation is likely to perceive that his or her organization actively performs CSR activities. Future research should consider this potential problem.

## 6. Conclusions

The aim of this paper was to reconcile mixed results from previous studies on the CSR–performance link by delving into the intermediating processes of the association. To empirically test these hypotheses, we used three-wave time-lagged data from South Korean workers. By performing a sequential mediation model analysis with an SEM technique, we demonstrated that the degree of organizational commitment of employees functions as a mediator in the association between CSR and organizational performance.

Although this paper has some limitations, this study stands to positively contribute to existing literature in the field of CSR by demonstrating an elaborate intermediating process and its contingent factor in the link. These findings emphasize the important role of micro-level mechanisms (i.e., OC and prosocial motivation) in the association between macro-level variables (i.e., CSR and OP). In addition, by demonstrating that prosocial motivation functions as a critical contextual factor to explain the CSR–OP link, we suggest and reveal that the sense-making process of employees (i.e., prosocial motivation) may critically influence the employee’s reactions toward CSR practices. In other words, this paper may contribute to resolving the mixed findings of previous research in the CSR–performance link.

## Figures and Tables

**Table 1:** Descriptive features of the sample.

Characteristic	Percent
Sex	
Male	48.3%
Female	51.7%
Age (years)	
20–29	20.9%
30–39	24.8%
40–49	26.8%
50–59	27.5%
Education	
Below high school	14.2%
Community college	19.5%
Bachelor’s degree	58.6%
Master’s degree or higher	7.6%
Work type	
Office workers	63.2%
Administrative positions	19.2%
Sales and marketing	5.9%
Manufacturing	5.3%
Education	1.7%
Others	4.6%
Position	
Staff	28.5%
Assistant manager	25.5%
Manager or deputy general manager	29.8%
Department/general manager or director and above	16.2%
Tenure (months)	
Below 50	52.3%
50–100	18.2%
100–150	15.6%
150–200	4.6%
200–250	4.3%
Above 250	5.0%
Firm size	
Fewer than 50 members	45.7%
50–99 members	12.3%
100–299 members	16.2%
300–499 members	7.0%
More than 500 members	18.9%
Industry Type	
Manufacturing	24.2%
Services	14.2%
Construction	12.9%
Information services and telecommunications	10.3%
Education	9.3%
Health and welfare	8.6%
Public service and administration	7.3%
Financial/insurance	4.0%
Others	9.2%

**Table 2:** Descriptive statistics. OC: organizational commitment.

	1	2	3	4	5	6	7	8
1. Position_T2	-							
2. Tenure (months) _T2	0.32 **	-						
3. Education_T2	0.14 *	−0.01	-					
4. Firm size_T2	−0.03	0.25 **	0.18 **	-				
5. Industry type_T2	0.02	0.04	0.07	−0.10	-			
6. CSR_T1	0.15 *	0.21 **	−0.02	0.23 **	0.03	-		
7. Prosocial Motivfation_T1	0.12 *	0.10	0.11	0.01	0.06	0.29 **	-	
8. OC_T2	0.25 *	0.19*	0.04	0.03	0.04	0.44 **	0.26 **	-
9. Organizational performance_T3	0.08	0.06	−0.11	0.02	0.14 *	0.33 **	0.12 *	0.47 **

Note: * *p* < 0.05. ** *p* < 0.01. T1, T2, and T3 means time point 1, 2, and, 3.

## Data Availability

No new data were created or analyzed in this study. Data sharing is not applicable to this article.

## References

[1] Li Z., Minor D.B., Wang J., Yu C. (2019). A learning curve of the market: Chasing alpha of socially responsible firms. J. Econ. Dyn. Contr..

[2] Lai C.S., Chiu C.J., Yang C.F., Pai D.P. (2010). The Effects of Corporate Social Responsibility on Brand Performance: The Mediating Effect of Industrial Brand Equity and Corporate Reputation. J. Bus. Ethics.

[3] Orlitzky M., Schmidt F.L., Rynes S.L. (2003). Corporate Social and Financial Performance: A Meta-Analysis. Organ. Stud..

[4] Saeidi S.P., Sofian S., Saeidi P., Saeidi S.P., Saaeidi S.A. (2015). How does corporate social responsibility contribute to firm financial performance? The mediating role of competitive advantage, reputation, and customer satisfaction. J. Bus. Res..

[5] McWilliams A., Siegel D. (2000). Corporate Social Responsibility and Financial Performance: Correlation or Misspecification?. Strateg. Manag. J..

[6] Lopez M., Garcia A., Rodriguez L. (2007). Sustainable development and corporate performance: A study based on the Dow Jones Sustainability Index. J. Bus. Ethics.

[7] Peng C.W., Yang M.L. (2014). The Effect of Corporate Social Performance on Financial Performance: The Moderating Effect of Ownership Concentration. J. Bus. Ethics.

[8] Gond J.-P., Moser C. (2021). Critical essay: The reconciliation of fraternal twins: Integrating the psychological and sociological approaches to ‘micro’ corporate social responsibility. Hum. Relat..

[9] Babu N., De Roeck K., Raineri N. (2020). Hypocritical organizations: Implications for employee social responsibility. J. Bus. Res..

[10] Aguinis H., Glavas A. (2019). On corporate social responsibility, sensemaking, and the search for meaningfulness through work. J. Manag..

[11] Aguinis H., Villamor I., Gabriel K.P. (2020). Understanding employee responses to COVID-19: A behavioral corporate social responsibility perspective. Manag. Res. J. Iberoam. Acad. Manag..

[12] Jones D.A., Newman A., Shao R., Cooke F.L. (2019). Advances in employee-focused micro-level research on corporate social responsibility: Situating new contributions within the current state of the literature. J. Bus. Ethics.

[13] Aguinis H., Glavas A. (2012). What we Know and Don’t Know about Corporate Social Responsibility: A Review and Research Agenda. J. Manag..

[14] Lin C., Chen S., Chiu C., Lee W. (2011). Understanding purchasing intention during product harm crises: Moderating effects of perceived corporate ability and corporate social responsibility. J. Bus. Ethics.

[15] Rupp D.E., Mallory D.B. (2015). Corporate social responsibility: Psychological, person-centric, and progressing. Annu. Rev. Organ. Psychol. Organ. Behav..

[16] Carroll A.B. (1999). Corporate social responsibility. Bus. Soc..

[17] Waddock S.A., Graves S.B. (1997). The Corporate Social Performance-Financial Performance Link. Strateg. Manag. J..

[18] Carmeli A., Gilat G., Waldman D.A. (2007). The role of perceived organizational performance in organizational identification, adjustment and job performance. J. Manag. Stud..

[19] Harrison D.A., Newman D.A., Roth P.L. (2006). How important are job attitudes? Meta-analytic comparisons of integrative behavioral outcomes and time sequences. Acad. Manag. J..

[20] Scott W.R. (1995). Institutions and Organizations.

[21] Allen N.J., Meyer J.P. (1990). The Measurement and Antecedents of Affective, Continuance and Normative Commitment to the Organization. J. Occup. Organ. Psychol..

[22] Meyer J.P., Allen N.J. (1997). Commitment in the Workplace: Theory, Research, and Application (Advanced Topics in Organizational Behavior).

[23] Brammer S., Millington A., Rayton B. (2007). The Contribution of Corporate Social Responsibility to Organizational Commitment. Int. J. Hum. Resour. Manag..

[24] Farooq O., Payaud M., Merunka D., Valette-Florence P. (2014). The impact of corporate social responsibility on organizational commitment: Exploring multiple mediation mechanisms. J. Bus. Ethics.

[25] Rego A., Leal S., Cunha M.P., Faria J., Pinho C. (2010). How the perceptions of five dimensions of corporate citizenship and their inter-inconsistencies predict affective commitment. J. Bus. Ethics.

[26] Stites J.P., Michael J.H. (2011). Organizational Commitment in Manufacturing Employees: Relationships with Corporate Social Performance. Bus. Soc..

[27] Turker D. (2009). How Corporate Social Responsibility Influences Organizational Commitment. J. Bus. Ethics.

[28] Whitener E.M., Brodt S.E., Korsgaard M.A., Jon M.W. (1998). Managers as initiators of trust: An exchange relationship framework for understanding managerial trustworthy behavior. Acad. Manag. Rev..

[29] Williams M. (2001). In whom we trust: Group membership as an affective context for trust development. Acad. Manag. Rev..

[30] Cooper-Hakim A., Viswesvaran C. (2005). The Construct of Work Commitment: Testing an Integrative Framework. Psychol. Bull..

[31] Mathieu J.E., Zajac D.M. (1990). A Review and Meta-Analysis of the Antecedents, Correlates, and Consequences of Organizational Commitment. Psychol. Bull..

[32] Meyer J.P., Stanley D.J., Herscovitch L., Topolnytsky L. (2002). Affective, Continuance, and Normative Commitment to the Organization: A Meta-Analysis of Antecedents, Correlates, and Consequences. J. Vocat. Behav..

[33] Riketta M. (2008). The causal relation between job attitudes and performance: A meta-analysis of panel studies. J. Appl. Psychol..

[34] Wright T.A., Bonett D.G. (2002). The moderating effects of employee tenure on the relation between organizational commitment and job performance: A meta-analysis. J. Appl. Psychol..

[35] Batson C.D., Berkowitz L. (1987). Prosocial motivation: Is it ever truly altruistic?. Advances in Experimental Social Psychology.

[36] Grant A.M. (2008). Does intrinsic motivation fuel the prosocial fire? Motivational synergy in predicting persistence, performance, and productivity. J. Appl. Psychol..

[37] Chatman J.A. (1989). Improving international organizational research: A model of person–organization fit. Acad. Manag. Rev..

[38] Kristof A.L. (1996). Person–organization fit: An integrative review of its conceptualizations, measurement, and implication. Pers. Psychol..

[39] Kristof-Brown A.L., Zimmerman R.D., Johnson E.C. (2005). Consequences of individuals’ fit at work: A meta-analysis of person–job, person–organization, person–group, and person–supervisor fit’. Pers. Psychol..

[40] Burt R.S. (1987). Social Contagion and Innovation: Cohesion versus Structural Equivalence. Am. J. Sociol..

[41] Wright P.M., Kehoe R.R., Klein H.J., Becker T.E., Meyer J.P. (2009). Organizational-level antecedents and consequences of commitment. Commitment in Organizations: Accumulated Wisdom and New Directions.

[42] Ostroff C. (1992). The relationship between satisfaction, attitudes, and performance: An organizational level analysis. J. Appl. Psychol..

[43] Wrzesniewski A., Dutton J.E., Debebe G. (2003). Interpersonal Sensemaking and the Meaning of Work. Res. Organ. Behav..

[44] Parker S.K., Atkins P.W., Axtell C.M., Hodgkinson G.P., Ford J.K. (2008). Building Better Workplaces through Individual Perspective Taking: A Fresh Look at a Fundamental Human Process. International Review of Industrial and Organizational Psychology.

[45] Latham G.P., Pinder C.C. (2005). Work motivation theory and research at the dawn of the twenty-first century. Annu. Rev. Psychol..

[46] Mitchell T.R., Daniels D., Borman W.C., Ilgen D.R., Klimoski R.J. (2003). Motivation. Handbook of Psychology, Vol. 12: Industrial and Organizational Psychology.

[47] Turker D. (2009). Measuring Corporate Social Responsibility: A Scale Development Study. J. Bus. Ethics.

[48] Kim B.J., Nurunnabi M., Kim T.H., Kim T.J. (2018). Doing Good Is Not Enough, You Should Have Been Authentic: The Mediating Effect of Organizational Identification, and Moderating Effect of Authentic Leadership between CSR and Performance. Sustainability.

[49] Grant A.M., Sumanth J.J. (2009). Mission possible? The performance of prosocially motivated employees depends on manager trustworthiness. J. Appl. Psychol..

[50] Jackson S.E., Joshi A., Erhardt N.L. (2003). Recent research on teams and organizational diversity: SWOT analysis and implications. J. Manag..

[51] Smith K.G., Smith K.A., Olian J.D., Sims H.P., O’Bannon D.P., Scully J.A. (1994). Top management team demography and process: The role of social integration and communication. Adm. Sci. Q..

[52] Anderson J.C., Gerbing D.W. (1988). Structural Equation Modeling in Practice: A Review and Recommended Two-Step Approach. Psychol. Bull..

[53] Browne M.W., Cudeck R. (1993). Alternative Ways of Assessing Model Fit.

[54] Shrout P.E., Bolger N. (2002). Mediation in Experimental and Nonexperimental Studies: New Procedures and Recommendations. Psychol. Methods.

[55] Brace N., Kemp R., Snelgar R. (2003). SPSS for Psychologists: A Guide to Data Analysis Using SPSS for Windows.

[56] Staw B.M. (1991). Dressing up like an organization: When psychological theories can explain organizational action. J. Manag..

[57] Eberle D., Berens G., Li T. (2013). The impact of interactive corporate social responsibility communication on corporate reputation. J. Bus. Ethics.

[58] Shin Y.H., Sung S.Y., Choi J.N., Kim M.S. (2014). Top management ethical leadership and firm performance: Mediating role of ethical and procedural justice climate. J. Bus. Ethics.

[59] Chun J.S., Shin Y., Choi J.N., Kim M.S. (2013). How does corporate ethics contribute to firm financial performance? The role of collective organizational commitment and organizational citizenship behavior. J. Manag..

[60] Arnold D.F., Bernardi R.A., Neidermeyer P.E., Schmee J. (2007). The effect of country and culture on perceptions of appropriate ethical actions prescribed by codes of conduct: A Western European perspective among accountants. J. Bus. Ethics.

[61] Eisenbeiss S.A. (2012). Re-thinking ethical leadership: An interdisciplinary integrative approach. Leadersh. Q..

[62] Hansen S.D., Dunford B.B., Boss A.D., Boss R.W., Angermeier I. (2011). Corporate social responsibility and the benefits of employee trust: A cross-disciplinary perspective. J. Bus. Ethics.

[63] Coles J.L., Li Z.F. (2019). An Empirical Assessment of Empirical Corporate Finance. https://ssrn.com/abstract=1787143.

[64] Li F., Morris T., Young B. (2019). The Effect of Corporate Visibility on Corporate Social Responsibility. Sustainability.

[65] Lee E.M., Park S.Y., Lee H.J. (2013). Employee perception of CSR activities: Its antecedents and consequences. J. Bus. Res..

[66] Collier J., Esteban R. (2007). Corporate social responsibility and employee commitment. Bus. Ethics Eur. Rev..

